# Characterization of the Key Aroma Compounds in Three Truffle Varieties from China by Flavoromics Approach

**DOI:** 10.3390/molecules24183305

**Published:** 2019-09-11

**Authors:** Tao Feng, Mengzhu Shui, Shiqing Song, Haining Zhuang, Min Sun, Lingyun Yao

**Affiliations:** 1 School of perfume and aroma technology, Shanghai Institute of Technology, No.100 Hai Quan Road, Shanghai 201418, China; 176071227@mail.sit.edu.cn (M.S.); sshqingg@163.com (S.S.); sunmin@sit.edu.cn (M.S.); Lyyao@sit.edu.cn (L.Y.); 2 Institute of Edible Fungi, Shanghai Academy of Agricultural Sciences, Key Laboratory of Edible Fungi Resources and Utilization (South), Ministry of Agriculture, National Engineering Research Center of Edible Fungi, 1000 Jinqi Road, Shanghai 201403, China; zhuanghaining@saas.sh.cn

**Keywords:** Yunnan Truffle, gas chromatography-olfactometry (GC-O), gas chromatography-mass spectrometry (GC-MS), flame photometric detector (FPD), odor-active volatiles (OAV), aroma recombination, flavoromics

## Abstract

The volatile compounds of three different fresh-picked truffle varieties (*Tuber sinensis*, T1, *Tuber sinoalbidum*, T2 and *Tuber sinoexcavatum*, T3) were extracted by headspace solid-phase microextraction (HS-SPME). Separation and identification of volatile components and sulfur compounds were investigated by gas chromatography-olfactometry (GC-O), gas chromatography-mass spectrometry (GC–MS) and gas chromatography with flame photometric detection (GC-FPD). The results showed that 44, 43 and 44 volatile compounds were detected in T1, T2 and T3 samples, respectively. In addition, 9, 10 and 9 sulfur compounds were identified in three samples by GC-FPD, respectively. Combining physicochemical and sensory properties, T1 presented fatty, green and rotten cabbage odor; T2 exhibited mushroom, sulfuric and musty odor notes; T3 had nutty, floral and roasted potato odor. Dimethyl sulfide, 3-methylbutanal, dimethyl disulfide, 3-octanone, bis(methylthio) methane, octanal, 1-octen-3-one, 1-octen-3-ol and benzeneacetaldehyde played indispensable roles in the overall aroma of three truffles. Finally, based on quantitative concentration in T1, odorous compounds (OAV) > 1 were mixed to recombine aroma, demonstrating that these key aroma compounds based on OAV can successfully recombine pretty similar aroma of each variety.

## 1. Introduction

Truffles (*Tuber* spp.) are ascomycete symbiotic fungi that strictly depend on other organisms to complete their life cycle [1]. High-quality truffles are mainly produced in limestone topographic areas, such as Alba (Italy), Perigo (France), Yunnan (China) etc. Especially the white Alba truffle (WAT, *Tuber pignatum pico*), is regarded as the noblest among the truffle species, because it elicits the most intense and pleasant aroma [2,3]. Truffles are very fastidious for the growth environment. As long as sunlight, humidity, soil pH value and the surrounding flora and fauna change slightly, they can’t grow. This is why the production of truffles is rare and the price is pretty expensive. Moreover, truffle also has many physiological activities, such as anti-virus, bacteriostasis, anti-inflammation, anti-cancer, anti-oxidation, liver protection and so on [4,5]. These physiological activities make truffle have great potential in the treatment of diseases. 

Aroma is one of the important factors to judge the quality of truffle [6]. Up to now more than 200 volatile substances have been reported in truffles [7]. However, not all flavoring substances contributed to the aroma of truffles. Whether these substances were sniffed depends on their own aroma concentration and threshold, of which the quotient leads to an odor activity value (OAV) to identify impact odorants [8,9]. Some sulfur compounds, though small in quantities, occupy an important position in truffle aroma. Different sulfur volatiles such as dimethyl sulfide, dimethyl disulfide, dimethyl trisulfide, and 3-(methylthio) propanal are similarly essential components of black European and Asian truffle aromas [10,11]. The most critical aroma compound in white truffle is bis (methylthio) methane (BMTM). Producers use BMTM as flavoring in truffle oil and various flavored truffle food products because it has highly effective olfactory characteristics [12]. 

Meanwhile, there have been more researches on the aroma of truffles. The volatile flavor compounds of black truffles were extracted by using simultaneous distillation-extraction (SDE) [13]. A total of 57 volatile compounds were identified, predominantly alcohols, ketones and aldehydes. Low boiling point volatile compounds were lost due to long high temperature cooking. Direct solvent extraction/solvent-assisted flavor evaporation (DSE-SAFE) coupled with a comprehensive two-dimensional gas chromatography (GC×GC) high resolution time-of-flight mass spectrometry (HR-TOF/MS) to compare the volatile compounds of Chinese black truffle (BT) and white truffle (WT) from Yunnan province. 3-methyl-butanoic acid, hexanoic acid, phenylethyl alcohol and 2-methyl-1-butanol were abundant in both BT and WT, whereas 2-methylpropanoic acid was only abundant in BT and benzyl benzoate in WT [14]. Research found the most important aroma compounds of black truffle (*Tuber melanosporum*) aroma were 2,3-butanedione, dimethyl disulfide (DMDS), ethyl butyrate, dimethyl sulfide (DMS), 3-methyl-1-butanol and 3-ethyl-5-methylphenol. In the case of summer truffle, the most important aroma molecules were DMS, DMDS, methional, 3-methyl-1-butanol, 1-hexen-3-one and 3-ethylphenol [15]. 

Flavoromics approach opens new perspectives for correlating the particular sensory attributes (odor properties) of food with its chemical composition [16]. The strategy involves a detailed profiling (fingerprinting) of volatile compounds, enabling a comparison between samples and the identification of key odorants by GC–O and GC–MS analyses [17]. Flavoromics was applied to find markers of cooked and fermented flavor in strawberry juices submitted to different treatments (heat, storage, and freeze-drying) [18]. Researchers also developed flavoromics to chemically profile the changes in a food product during aging to provide a unique basis to investigate changes in flavor profiles, identifying chemical attributes that may relate to freshness perception in food [19].

The output and exports of truffles in China are increasing year by year, but there is a lack of thorough and comprehensive flavor research on different varieties of truffles, especially the correlation of their odor-active compounds under a multivariate analysis. Therefore, the aim of the current study were to (1) identify the odor-active compounds in truffles by GC-O from three aspects: Aroma description, aroma intensity and frequency, (2) analyze the volatile compounds by GC-MS and FPD via polar, non-polar column and external standard quantitative method accurately, (3) calculate OAVs of volatile compounds and sensory evaluation of three truffle samples, finding aroma fingerprinting of each species (4) to confirm the aroma contribution from screened out high OAV compounds by aroma recombination. Therefore, the key aroma compounds in three truffle varieties from China would be characterized by flavoromics approach.

## 2. Results and Discussion

### 2.1. GC-O Results for Truffles

The aroma substances of three kinds of fresh truffles were extracted by HS-SPME and characterized by GC-O. The results were summarized in Table 1, which listed 38, 37 and 38 odor-active compounds corresponding to T1, T2 and T3 truffle samples, respectively. Three samples were characterized by comparison with their retention indices (RIs), aroma description and mass spectra with authentic standards. The aroma intensities (AIs) of volatile components ranged from 1.2 to 8.7 for T1, 1.2 to 9.2 for T2, 1.1 to 8.8 for T3.

In T1 sample, dimethyl sulfide (8.7), dimethyl disulfide (8.5), octanal (8.4) and 1-octen-3-one (8.3); in T2 sample, dimethyl sulfide (8.6), 3-octanone (8.9), bis(methylthio) methane (8.4), 1-octen-3-one (8.8), 3-octanol (8.6) and 1-octen-3-ol (9.2); in T3 sample, dimethyl sulfide (8.3), 2-methyl-butanal (8.8), 2-methylbutanol (8.1), 3-(methylthio)propanal (8.4) and benzeneacetaldehyde (8.3) had relatively higher aroma intensities (AIs).

As was shown in the Table 1, the aroma frequencies of ten panelists during GC-O experiment were presented. Dimethyl sulfide, 2-methyl-butanal, 3-methyl-butanal, hexanal, heptenal, 2-methylbutanol, 2-pentyl-furan, 3-octanone, dimethyl disulfide, bis(methylthio) methane, 2-octanone, octanal, 1-octen-3-one, 3-octanol, 1-octen-3-ol, 3-(methylthio)propanal, benzeneacetaldehyde had relatively higher frequency than other compounds. There was no doubt that most of these higher frequency components had high AIs simultaneously. Thus, the method through combining AIs with frequency of volatiles probably could find out potential important compounds in truffle [21,22].

From Table 1, the three samples differed greatly in sensory olfaction. Dimethyl sulfide and dimethyl disulfide had high AIs in T1 sample, which brought more decayed cabbage odor and sweet smell of popcorn (Table 1). Both of the compounds were also considered to represent the aroma of black truffles [23]. Eight carbon volatile compounds accounted for large proportion of aroma compounds in T2 samples, including 3-octanone, 2-octanone, 1-octen-3-one, 3-octanol, 1-octen-3-ol. Eight carbon alcohol and ketone mainly provided aroma of mushroom, earthy and herbal. These compounds had been found in most fungi, which gave samples typical mushroom odors [24,25]. Bis(methylthio)methane has always been considered as the most important sulfide in Italian white truffle (*Tuber magnatum Pico*) research [26] and it has also been found to play an important role in T2 samples. In addition to sulfur compounds, 2-methyl-butanal and 3-methyl-butanal were studied as important aroma compounds in different truffles [27,28], however, T3 samples had much less rough sulfide odor and more focused on 2-methyl-butanal and 3-methyl-butanal, which provided strong nutty and grain aroma. The AI of phenylacetaldehyde was also very high in T3 sample.

### 2.2. Quantitative Analysis and OAV of Volatile Compounds 

As was shown in Table 2, a total of 44, 43 and 44 substances were detected in T1, T2 and T3, respectively. T1 included 13 alcohols, 9 nitrogen or sulfur-containing compounds, 11 aldehydes, 3 ketones, 3 esters, 2 acids, 2 hydrocarbons and 1 ester; T2 included 12 alcohols, 10 nitrogen or sulfur-containing compounds, 11 aldehydes, 3 ketones, 3 esters, 2 acids and 2 hydrocarbons; T3 included 12 alcohols, 9 nitrogen or sulfur-containing compounds, 14 aldehydes, 3 ketones, 2 esters, 2 acids and 2 hydrocarbons. 

Quantitative analysis based on external standard method (Table 3), the flavor substances with higher content (μg/kg) in T1 were as the following: dimethyl sulfide (1260), dimethyl disulfide (1139), 3-methylbutanal (2187), octanal (873), P-cresyl methyl ether (870.84) and 2-methylbutanol (734). Except for the characteristic aromatic substances of black truffle, T1 also contained a large amount of p-cresol methyl ether, which made the black truffles produced in Nanhua Yunnan have the fragrance [31] of Ylang Ylang(*Cananga odorata (Lamk.) Hook.*) and violet (*Matthiola incana (L.) R. Br*.

The major volatile compounds in T2 were dimethyl sulfide (1156), 2-methylbutanol (1123), 3-octanone (6300), 1-octen-3-one (1034), 3-octanol (2157), 1-octen-3-ol (5849). Nevertheless, 2, 4-dithiopentane, a typical aroma in Italy famous white truffle *Tuber magnaturn* [32], was not found in Chinese white truffle. 

In T3, dimethyl sulfide (1089), 2-methylbutanal (580), 3-methylbutanal (4573), 2-methylbutanol (3879), 3-octanone (950), octanal (436), 1-octen-3-ol (566) and benzeneacetaldehyde (403), had higher concentration. This result was similar to another major truffle species *Tuber indicum* in China. Researchers also found that the highest content compound was dimethyl sulfide, followed by 3-methylbutanal, 2-methylbutanal, 2-butanol and 1-octen-3-ol in *Tuber indicum* [25].

Of the above-mentioned compounds, the same characteristic compound of three samples was dimethyl sulfide, which has been described as responsible for the detection of such fungi by animals, such as wild boar and trained dog [33]; it had also been detected in different species of truffles, such as *T. melanosporum*, in which it was also the only quantitatively important sulfur volatile organic compound (VOC) [22,34,35]. Simultaneously, these compounds had high AIs and OAVs, which could be regarded as active-odor compounds in truffles. From Table 3, volatile components like dimethyl sulfide (3630–4200), 3-methylbutanal (15–508), 3-octanone (672–4846), octanal (61–1247), 1-octen-3-one (102–1293), 1-octen-3-ol (437–5849), benzeneacetaldehyde (34–576), dimethyl disulfide (3–163) bis(2-methyl-3-furyl)disulfide (129) showed relatively higher OAVs than other compounds, indicating that critical influence to the aroma of truffle. Although some compounds were present at relatively low concentration (<100 μg/kg), the OAVs were above 1 because of their lower thresholds, such as hexanal (33.56–53.63 μg/kg), (*E*)-2-nonenal (8.76–23.59 μg/kg), (*E*)-2-octenal(7.68 μg/kg), nonanal(38.75–65.38 μg/kg), bis(methylthio)methane (273–658), 3-methyl butanoic acid (6.32–9.45 μg/kg), limonene(3.61–45.79 μg/kg), 4-isopropyltoluene(4.81 μg/kg), so these compounds also played significant roles in the aroma of truffle.

The compounds had high OAVs showed that the aroma of the three varieties of Chinese truffles were less strong, pungent sulfurous odor, plenty of sweetness of flowers and fatty notes. This conclusion also had many similarities with other related Chinese truffle aroma studies [15,26]. The unique aroma of Chinese truffles might also be related to the plant environment, soil conditions, such as host tree and complex bacterial colonies in soils [36,37]. GC-O sensory evaluation combined with OAVs could provide a better assessment of key aroma compounds.

### 2.3. Sensory Analysis

**Figure 1:**
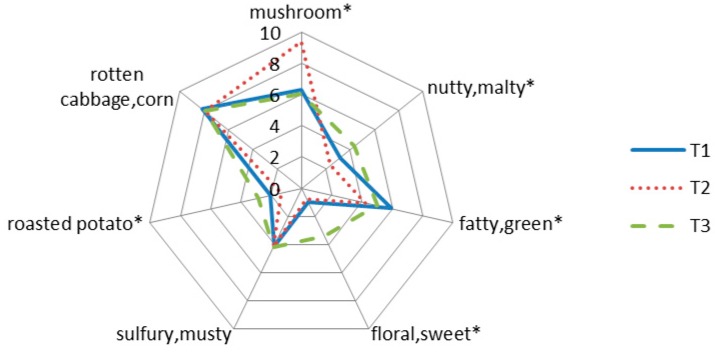
The aroma profiles of truffle samples obtained from T1, T2 and T3 samples. The seven notes with “*” are significantly different between samples (*p* < 0.05).

After consensus session of sensory evaluation, seven notes were selected to evaluate the aroma of three truffle samples by well-trained panelists, following “mushroom”, “nutty and malty”, “fatty and green”, “floral and sweet”, “sulfuric and musty”, “roasted potato”, “rotten cabbage and corn”. ANOVA statistical analysis showed that “mushroom”, “nutty and malty’, “fatty and green”, “floral and sweet”, “roasted potato” notes were significantly different in three truffle samples (*p* < 0.05) (Figure 1) through sensory evaluation scores (Table 4).

**Figure 2:**
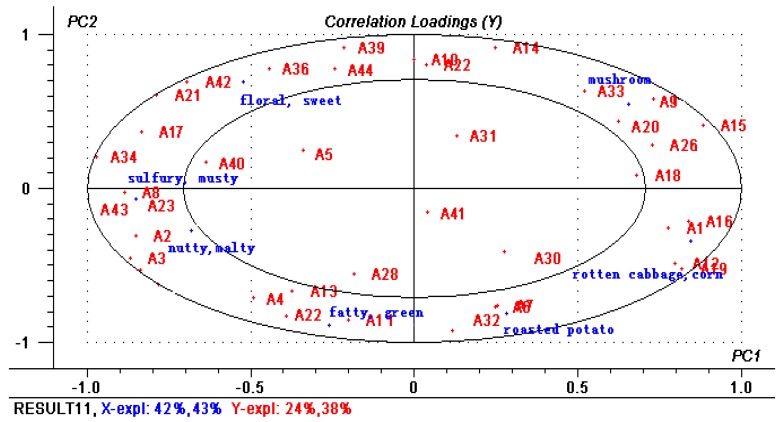
The model of PLS2 was derived from GC-O characterized compounds as the X-matrix and flavor notes as Y-matrix, respectively. Volatile compounds of **A1**–**A44** correspond to the code compounds in Table 1.

PLSR was applied to certify the correlation between the GC-O data and flavor notes by the panelists. The 44 odor-active compounds characterized by GC-O were used as X-matrix, and the 7 flavor notes obtained by sensory evaluation were Y-matrix, which generated the correlation load diagram of PLSR as shown in Figure 2. The two ellipses represent the variance contribution rate of 50% (small) and 100% (large), respectively. The volatiles between the two ellipses had a good correlation with the seven flavor notes. As could be seen from Figure 2, the contribution rate of X variable was 85%, and that of Y variable was 62%, indicating that the PLSR model can further verify the correlation between volatile compounds and flavor notes. The specific analysis was as the following:

T1 sample was thought to have much more fatty, green and rotten cabbage, corn aroma than other two samples. From Table 1, AIs of hexanal (**A5**), octanal (**A19**), nonanal (**A22**) in T1 were higher than those of T2, T3 and flavor note of these compounds were closely related to the “green, fatty, and waxy”; Dimethyl sulfide and 2-acetylthiazole had high AIs and concentrations in T1, which contributed to the rotten cabbage, corn notes (Figure 2).

In T2 sample, mushroom and sulfuric, musty attributes were clearly detected. 3-octanone (**A15**) and 1-octen-3-ol (**A31**) had both higher concentration and OAV than other two samples, which provided typical mushroom aroma (Figure 2). According to GC-O, bis(methylthio)methane (**A17**) was regarded to contribute rough onion and garlic flavor.

T3 sample was much more described as “nutty and malty”, “floral and sweet”, “roasted potato” than other two samples. 2-methylbutanal (**A2**) and 3-methylbutanal (**A3**) were thought to relate to “nutty and malty”. Benzeneacetaldehyde (**A39**) and phenylethyl alcohol (**A44**) had sweet rose-like fragrance. The note of “roasted potato” mainly came from 3-methylthiopropanal (**A32**).

### 2.4. Aroma Recombination

**Figure 3:**
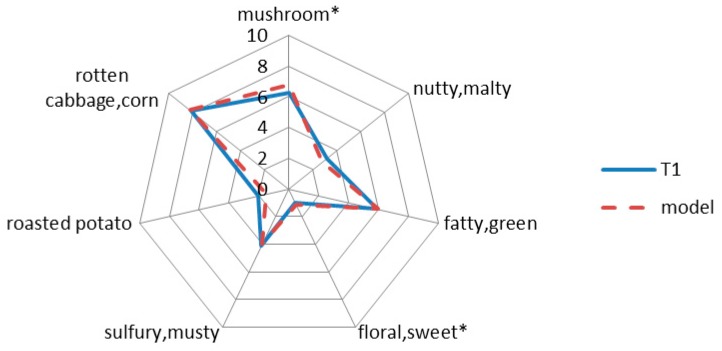
Comparative aroma profiles of T1sample and the aroma recombination model. The seven descriptors with “*” are significantly different between T1 and model. (*p* < 0.05).

Aroma recombination model was performed to verify the quantitative data and selected key compounds (OAV > 1). Model was evaluated by panelists with original sample T1 according to seven flavor notes. As was shown in Figure 3, “nutty and malty”, “roasted potato” of model presented lower score than T1 sample, while “mushroom” note of model was higher than that of T1 sample. From the general flavor profile, model and T1 sample did not have significant differences by seven notes, demonstrating that these key aroma compounds based on OAV could successfully recombine pretty similar aroma. 

## 3. Materials and Methods

### 3.1. Chemicals

2-methylbutanal, 3-methylbutanal, pentanal, isopropyl alcohol, 1-propanol, 1-butanol, hexanal, 2-methyl-1-propanol, (*E*)-2-methyl-2-butenal, limonene, heptenal, 2-butenal, 2-methylbutanol, 3-methylbutanol, 2-pentyl-furan, 3-octanone, 1-pentanol, 2-octanone, 4-isopropyltoluene, octanal, 1-octen-3-one, isobutyl hexanoate, nonanal, heptanoic acid ethyl ester, 1-hexanol, 3-octanol, octanoic acid ethyl ester, (*E*)-2-octenal, (*E*)-2-nonenal, P-cresyl methyl ether, 1-heptanol, 1-octen-3-ol, 1-octanol, benzaldehyde, 2-methyl-butanoic acid, 3-methyl-butanoic acid, 2-acetylthiazole, benzeneacetaldehyde, benzyl alcohol, phenylethyl alcohol, γ-nonalactone, were purchased from Alfa Aesar Corporation (Tianjin, China). Methanethiol, dimethyl sulfide, dimethyl disulfide, bis(methylthio)methane, 3-(methylthio)propanal, dimethyl sulfoxide, dipropyl trisulfide, 3-(methylthio)propanol, dimethyl sulfone, bis(2-methyl-3-furyl)disulfide were purchased from Sigma-Aldrich (St Louis, MO, USA). All of the chemical standards used above were of GC quality.

### 3.2. Materials

Three varieties of ripe truffle namely black truffle *Tuber sinensis* (T1), white truffle *Tuber sinoalbidum* (T2) and *Tuber sinoexcavatum* (T3), were collected from Nanhua County, Yi Autonomous Prefecture, Yunnan Province of China at December 19th and 20th, 2018. After collection, they were wrapped in non-woven fabrics and transported to the laboratory with the ice bag within 24h. The fresh truffle samples were washed with Milli-Q water and crushed into truffle purees via JYL-C051 type blender (Joyoung, Shandong, China), and kept in the −18 °C refrigerator for further study.

### 3.3. Solid Phase Microextraction (SPME) Absorption of Aroma Compounds

5.0 g fresh truffle purees were accurately weighed in 20 mL vials, Teflon covers and added 5 μL internal standard solutions (100 mg/L 1, 2-dichlorobenzene or 100 mg/L 2-methyl-3 -tetrahydrofuran thiol for sulfide). Samples were kept at 45 °C in a water bath with 10 min of equilibration time. 

A 50/30 μm divinylbenzene-Carboxen-polydimethylsiloxane (DVB-CAR-PDMS) fiber (Supelco, Bellefonte, PA, USA) with a 1 cm length was used. The extraction time was 45 min. Before chemical absorption, the fiber was preconditioned for 30 min on an Agilent 7890 gas chromatograph (Agilent Technologies, Santa Clara, CA, USA) with the injector temperature of 250 °C. 

### 3.4. SPME-GC-FID-O Analysis of Truffle

The Agilent 7890A gas chromatograph was used for GC-O analysis. The gas chromatograph was equipped with flame ionization detector (FID) and ODP-2 olfactory port (Gerstel, Mulheim an der Ruhr, Germany). GC effluent was split into 1:1 between the FID and sniffing port. Purified, moist air flowing with odorant eluting were carried to the individual olfactory assessor via an insulated stainless steel tube at 40 mL/min. Samples were conducted using a HP-Innowax and a DB-5 analytical fused silica capillary column (both columns: 60 m × 0.25 mm × 0.25 μm; Agilent Technologies, Santa Clara, CA, USA). GC-FID-O analysis conditions were as the following: the flow rate of carrier gas (nitrogen) was 1.8 mL/min, the oven temperature was held at 50 °C, ramped with a rate of 10 °C/min to 100 °C, and then ramped to 140 °C at a rate of 3 °C/min, finally reached at 200 °C with a rate of 2 °C/min and kept for 10 min. The injection mode was set in splitless for 3 min at 250 °C. The desorption time was 5 min.

The olfactory experiment was performed by 10 trained panelists (six females and four males). Panelists were very sensitive to aroma identification by training olfactory characteristics of reference compounds and truffle sample matrices in sniffing bottles. Aroma characteristics, aroma intensity and frequency of occurrence were written down by the assessors with 50 min of sniffing time. The intensity was calculated as the average of all panelists’ scores for an identified aroma. The odor intensities were evaluated on a 10-point intensity scale, where 0 meant a compound had no odor, 5 represented a moderate intensity and 10 stood for an extreme strong odor. Each sample was performed in triplicate by each panelist.

### 3.5. Calibration of Standard Curves

Similar truffle matrix was prepared by adding 2.4 mg/g glucose, 48 mg/g mannitol, 5.2 mg/g malic acid, 2.4 mg/g alanine, 3.7 mg/g glutamate and 1.8 mg/g glycine in Milli-Q deionized water before external standard quantification [38,39]. A recombination containing all volatile compounds was diluted with methanol to 1:5, 1:10, 1:20, 1:50, 1:100 and 1:200 strengths. Then, 5 μL 1,2-dichlorobenzene (100 mg/L) was introduced to the 5 g of model matrix in a 20 mL vial to establish the calibration curves. Equally, 5 μL 2-methyltetrahydrofuran-3-thiol (100 mg/L) was added to establish the calibration curves for sulfur compounds. These mixture models were extracted by HS−SPME. The standard curves, coefficient of determination (r^2^) and validated linear range for the volatile compounds were set up. All experiments were repeated three times. The calculation formula was as the following:

(*A_x_*/*A_i_*) = *a* (*C_x_*/*C_i_*) + *b* (1)

(*A_x_/A_i_*) equaled peak area of volatile compounds standard/peak area of internal standard; (*C_x_/C_i_*) represented concentration of volatile compounds standard/concentration of internal standard; *a* is the slope and b is the intercept on y axis of the standard curve. 

### 3.6. SPME-GC–MS of Volatile Compounds in Truffle

The volatile compounds were analyzed by an Agilent 6890 gas chromatography with SPME and a 5975 mass selective detector (MSD) (Agilent Technologies, Santa Clara, CA, USA), HP-Innowax and DB-5 analytical fused silica capillary column (both columns: 60 m × 0.25 mm × 0.25 μm; Agilent, Santa Clara, CA, USA). Conditions for GC-MS analysis were as the following: the injection port was set in a splitless mode, and the desorption time was 5 min and the desorption temperature was 250 °C, the temperature program referred as that of GC-O, the carrier gas was helium with a constant flow rate of 1 mL/min. Chemical identification was performed by MSD. Its electron ionization energy was 70 eV. The temperature of ion source was set at 230 °C. The compounds were identified by matching retention time of authentic standards, retention indices (RIs), and mass spectra in the NIST 11 database. RIs in the literature that matched the column condition (60 m × 0.25 mm × 0.25 μm) and temperature ramp were compared, according to the Van Den Dool and Kratz RI. The RIs of unknown compounds were determined by pure n-alkanes mixture (C_5_−C_30_, Sigma-Aldrich, St. Louis, MO, USA). The calculation formula was as the following:

(2) $$RI_{x}=(\frac{\mathrm{lg}(t_{x})-\mathrm{lg}(t_{z})}{\mathrm{lg}(t_{z+1})-\mathrm{lg}(t_{z})}+Z)\times 100$$

*t_x_* represented the retention time of volatile compounds; *t_z_* was the retention time of n-alkanes which had same carbon atoms of volatile compounds; *Z* was the number of carbon atoms of volatile compounds. The GC-MS chromatograms are given in the Supplementary Materials. 

### 3.7. SPME-GC-FPD Detection of Sulfur Containing Volatile Compounds in Truffle

The Agilent 7890A gas chromatography equipped with FPD was used to detect sulfur compounds in truffle samples. The oven temperature and heating procedure were consistent with the setting of GC-MS. The FPD temperature was set at 250 °C, the PMT voltage was set at 500 V. The desorption time was 5 min and the injection mode was splitless. The sulfur-containing compounds were identified by retention time of authentic standards and retention index on both columns.

### 3.8. Odor Activity Value (OAV)

By using the formula of olfactory activity value, OAV = C/T, in which OAV represents the olfactory activity value of the flavor compound, C represents the concentration of each compound, T represents the detection threshold in air. Available threshold values were from literature reference. It is generally believed that aromatic compounds with high OAV are most likely to be the key contributors to the overall aroma. OAV > 1 indicates that the compound has a direct impact on the aroma [40]. 

### 3.9. Sensory Evaluation

According to the guidelines and conditions of ISO 8589-2007, sensory evaluation was carried out in the sensory laboratory. Based on the previous studies [2,41], the method of sensory analysis was generic descriptive analysis. 20 g truffle puree in the 100 mL plastic cup was prepared with a Teflon cover for evaluation. At the beginning, the aroma of truffles was evaluated by a well-trained panel of 10 members (4 males and 6 females). Then through the three preliminary consensus training (each 2 h), eventually the panelists made the final agreement about the aroma description of truffle (“sulfuric, musty”, “rotten cabbage, corn”, “nutty, malty”, “roasted potato”, “fatty, green”, “mushroom-like” and “floral, sweet” note). Each sensory attribute was defined as following reference compounds: bis(methylthio)methane for “sulfuric, musty” note, dimethyl sulfide for “rotten cabbage, corn” note, 3-methylbutanal for “nutty, malty” note, 3-(methylthio)propanal for “roasted potato” note, octanal for “fatty, green” note, 1-octen-3-ol for “mushroom-like” note, benzeneacetaldehyde for “floral, sweet” note. 

A 0-10 linear scale, from 0 (not perceivable) to 3.0, 4.0, 5.0 (moderately perceivable) to 10.0 (very strongly perceivable) were given to the intensities of the respective aroma qualities. The sensory evaluation experiment of each sample was repeated three times to find the average value.

### 3.10. Aroma Recombination of Truffle

Black truffle T1 sample is very popular and has high sales share in China market, so it was chosen to make aroma recombination. Accordingly, in order to confirm high OAV compounds play important roles in T1′s aroma, a total of 21 (OAV ≥ 1) volatile compounds were added in an aqueous solution aroma model at their actual quantitative concentration as determined in T1. The sensory panelists scored the aroma recombination model and truffle samples through seven sensory notes discussed in generic descriptive analysis. Each sample was evaluated in triplicate by each member.

### 3.11. Statistical Analysis

In this study, relative standard deviation (RSD) well reflected the precision of GC-MS and GC-O test data. Aroma intensity of GC-O and quantitative of volatile compounds were performed by analysis of variance (ANOVA). When there were significant differences between samples, Duncan’s multiple range tests were used at the level of significance set at *p* < 0.05. Both ANOVA and Duncan’s multiple range tests were conducted by Predictive Analytics Software (PASW) Statistics 18 (IBM, Chicago, IL, USA). 

Partial least squares regression (PLSR) analysis was carried out by the Unscrambler 9.8 (CAMO ASA, Oslo, Norway). Odor-active compounds detected with GC-O as X-variables and the odor attributes from the descriptive profile as Y-variables. The correlation between GC-O data and sensory attributes was illustrated by PLS2. All regression models were validated using full cross-validation.

## 4. Conclusions

In summary, the volatile constituents of truffles from three different varieties from Yunnan Province of China were studied by flavoromics approach through SPME extraction combined with GC-O, GC-MS, GC-FPD and aroma recombination. 44, 43 and 44 volatile compounds were detected in T1, T2 and T3 samples, respectively. Among them, 9, 10, and 9 sulfur-containing compounds were authenticated. Dimethyl sulfide, 3-methylbutanal, 2-methylbutanol, 3-octanone, 1-octen-3-one, 3-octanol and1-octen-3-ol showed higher level in external standard quantitative analysis. Finally, 24 key aroma-active compounds screened out by GC-O and OAV were considered to make important contribution to the overall aroma of three truffles. 

Although there were some differences in the aroma of the three Chinese truffles, in general, the aroma of Chinese truffles was more floral, mushroom and sweet, which was also the unique aroma of Chinese Yunnan truffles. Through a flavoromics study on the fragrance of domestic truffles, further development of truffle products with Chinese characteristics, and transformation of the Chinese truffle market from original excavation to intensive processing industry.

## Figures and Tables

**Table 1:** GC-O identified aroma-active compounds in truffle samples.

No.	Compound ^A^	RI (Calculate)		RI (Reference) ^B^		Aroma	Identification ^D^	Aroma Intensity						Frequency ^F^		
		DB-5	HP-Innowax	DB-5	HP-Innowax	Description		T1	RSD (%)	T2	RSD (%)	T3	RSD (%)	T1	T2	T3
**A1**	Dimethyl sulfide	515	721	515	716	sulfuric, garlic, cabbage-like	AD, RI, Std	8.7a ^E^	6.4	8.6a	5.3	8.3a	6.8	10	10	10
**A2**	2-Methylbutanal	641	923	640	925	cocoa, almond-like	AD, RI, Std	8.3a	6.8	7.7b	5.2	8.5a	5.1	10	10	10
**A3**	3-Methylbutanal	650	913	650	910	green, nutty, cocoa-like	AD, RI, Std	7.6ab	5.6	6.6b	4.9	8.8a	3.4	10	10	10
**A4**	Pentanal	715	935	732	935	vegetable, green	AD, RI, Std	5.5a	7.1	5.8a	6.5	5.2a	6.7	8	9	8
**A5**	Hexanal	802	1084	801	1084	grass, leafy, fruity, sweaty	AD, RI, Std	5.2ab	6.8	6.8a	4.9	4.6b	5.9	10	10	9
**A6**	Dimethyl disulfide	785	1086	785	1086	sulfuric, cabbage, onion-like	AD, RI, Std	8.5a	5.9	7.3b	5.1	6.9b	4.2	10	10	10
**A7**	2-Methylpropanol	608	1094	609	1098	winey	AD, RI, Std	2.2	15.2	—	—	1.8	27.9	8	0	7
**A8**	(*E*)-2-Methyl-2-butenal	743	1089	739	1088	fruity, green, almond, nutty	AD, RI, Std	3.1b	5.9	3.3b	7.2	4.9a	6.2	8	8	8
**A9**	Limonene	1031	1192	1031	1198	citrus, orange, fresh, sweet	AD, RI, Std	4.8a	6.7	3.5b	7.3	3.2b	5.8	9	9	9
**A10**	(*E*)-2-Heptenal	960	1334	961	1336	fresh, aldehydic, fatty, green	AD, RI, Std	7.8a	5.3	4.5b	6.6	7.2a	5.4	10	10	10
**A11**	(E)-2-Butenal	646	1050	644	1047	green, vegetable	AD, RI, Std	—	—	—	—	3.3	8.1	0	0	8
**A12**	2-Methylbutanol	742	1206	742	1208	malty	AD, RI, Std	3.8b	7.9	4.3b	8.3	8.1a	5.8	10	10	10
**A13**	3-Methylbutanol	736	1209	732	1209	roasted, winey, onion-like	AD, RI, Std	4.1b	5.8	4.6ab	5.2	4.8a	6.9	9	8	7
**A14**	2-Pentyl-furan	994	1239	994	1235	fruity, green, earthy	AD, RI, Std	5.6a	6.7	4.5b	6.3	4.1b	7.2	10	10	10
**A15**	3-Octanone	984	1243	984	1240	herbal, lavender, mushroom	AD, RI, Std	7.6b	6.9	8.9a	5.9	7.7b	6.1	10	10	10
**A16**	1-Pentanol	763	1262	763	1256	fusel	AD, RI, Std	2.2a	8.1	1.2b	28.8	1.1b	25.6	8	6	6
**A17**	Bis(methylthio) methane	898	1280	896	1282	sulfuric, garlic	AD, RI, Std	8.3a	5.3	8.4a	4.1	7.1b	9.6	10	10	10
**A18**	2-Octanone	965	1244	965	1244	earthy, herbal	AD, RI, Std	7.1a	5.8	7.5a	5.7	7.3a	4.5	10	10	10
**A19**	Octanal	1006	1282	1006	1280	waxy, orange, peel	AD, RI, Std	8.4a	5.9	4.8c	6.3	6.2b	8.9	10	9	10
**A20**	1-Octen-3-one	975	1305	975	1305	mushroom, earthy, musty	AD, RI, Std	8.3a	6.2	8.8a	7.5	6.1b	7.4	10	10	10
**A21**	Isobutyl hexanoate	1143	1344	1145	1356	fruity, pineapple, green	AD, RI, Std	5.4	6.3	—	—	—	—	9	0	0
**A22**	Nonanal	1104	1358	1104	1358	waxy, aldehydic, fatty	AD, RI, Std	6.5	7.7	—	—	5.8	6.5	10	0	9
**A23**	Heptanoic acid ethyl ester	1089	1329	1093	1329	fruity, pineapple, banana, strawberry	AD, RI, Std	3.5a	5.1	3.3ab	8.1	2.8b	15.3	8	7	8
**A24**	1-Hexanol	864	1364	851	1360	alcoholic, pungent, green	AD, RI, Std	1.9a	5.8	1.8a	26.5	2.2a	17.4	9	9	9
**A25**	Unknown 1	— ^C^	1396	—	—	pungent	AD	1.2	18.2	—	—	2.9	17.9	7	0	8
**A26**	3-Octanol	991	1386	995	1386	earthy, mushroom, herbal	AD, RI, Std	7.6b	5.8	8.6a	6.2	7.3b	9.6	10	10	10
**A27**	Octanoic acid ethyl ester	1196	1442	1196	1446	fruity, creamy, mushroom	AD, RI, Std	—	—	5.9	6.7	—	—	0	8	0
**A28**	(*E*)-2-Octenal	1057	1432	1057	1434	green, citrus, peel, fatty	AD, RI, Std	—	—	5.2	7.2	6.1	3.2	0	9	10
**A29**	2-Nonenal	1161	1536	1161	1537	green, cucumber, fatty	AD, RI, Std	4.6a	5.6	4.9a	5.9	3.8b	15.5	9	8	9
**A30**	Heptanol	972	1457	972	1457	musty, sweet, woody	AD, RI, Std	2.1	17.5	1.9	25.2	—	—	8	8	0
**A31**	1-Octen-3-ol	982	1426	982	1426	mushroom, earthy	AD, RI, Std	7.8b	6.3	9.2a	6.4	8.1b	9.6	10	10	10
**A32**	3-(Methylthio)propanal	907	1456	907	1456	musty, potato, onion, beefy,	AD, RI, Std	7.9a	5.9	7.7a	5.9	8.4a	8.9	10	10	10
**A33**	1-Octanol	1068	1564	1068	1564	waxy, green, citrus	AD, RI, Std	3.8a	7.9	3.7a	8.5	3.3a	6.4	8	8	6
**A34**	Dimethyl sulfoxide	825	1560	820	1560	cheesy, garlic, mushroom	AD, RI, Std	—	—	—	—	5.5	9.6	0	0	10
**A35**	Unknown 2	—	1598	—	—	smoky	AD	3.9	6.7	4.8	7.6	—	—	8	9	0
**A36**	Benzaldehyde	963	1528	963	1528	sweet, bitter, almond, cherry	AD, RI, Std	5.3a	6.5	2.6b	14.8	5.8a	6.2	9	10	9
**A37**	2-Methylbutanoic acid	858	1650	858	1652	acid, fatty	AD, RI, Std	4.5a	5.6	2.7b	17.3	2.1b	18.2	8	7	7
**A38**	3-Methylbutanoic acid	846	1684	875	1686	pungent, acid, cheese	AD, RI, Std	3.8ab	8.4	3.2b	6.3	4.2a	6.9	9	8	9
**A39**	Benzeneacetaldehyde	1051	1646	1051	1646	honey, sweet, floral	AD, RI, Std	5.2b	6.9	4.8b	6.5	8.3a	4.8	10	10	10
**A40**	3-(Methylthio)propanol	998	1706	998	1706	sulfuric, onion, garlic	AD, RI, Std	5.8b	7.8	6.2a	7.8	6.5a	7.4	9	8	9
**A41**	Unknown 3	—	1831	—	—	sulfuric	AD	4.8	6.2	—	—	3.7	7.9	8	0	8
**A42**	Benzyl alcohol	1038	1890	1035	1886	floral, rose, balsamic	AD, RI, Std	—	—	2.1	14.9	—	—	0	8	0
**A43**	Dimethyl sulfone	913	1912	925	1912	sulfuric	AD, RI, Std	—	—	7.3	5.6	—	—	0	10	0
**A44**	Phenylethyl alcohol	1110	1923	1113	1923	floral, rose	AD, RI, Std	3.3c	8.4	4.5b	6.7	6.7a	6.1	7	6	9

^A^ Volatile compounds detected in truffle samples; ^B^ Retention index of compounds on DB-5 and HP-Innowax columns [20]; ^C^ not detected; ^D^ RI: retention index; Std: confirmed by the authentic standard; AD: Aroma descriptor; ^E^ Values with different roman letters (a–c) in the same row are significantly different according to the Duncan test (*p* < 0.05); ^F^ Aroma frequency by sensory panelist.

**Table 2:** Compounds detected in truffle samples by GC-MS and GC-FPD.

No.	Compounds ^A^	Identification ^B^	RI (calculate)		RI (reference) ^C^		Concentration (μg kg^−1^)						Threshold ^G^	OAV ^H^		
			DB-5	HP-Innowax	DB-5	HP-Innowax	T1	RSD (%)	T2	RSD (%)	T3	RSD (%)	(μg kg^−1^)	T1	T2	T3
**1**	Methanethiol	FPD,RI,Std	464	696	464	690	7.52	1.84	4.65	2.68	—	—	4	2	1	—
**2**	Dimethyl sulfide	FPD,RI,Std	515	721	515	716	1260a ^D^	87.12	1156a	66.29	1089a	59.73	0.3	4200	3853	3630
**3**	2-Methylbutanal	MS,RI,Std	641	923	640	925	335ab	35.45	189c	17.4	580a	43.28	12.5	27	15	46
**4**	3-Methylbutanal	MS,RI,Std	650	913	650	910	2781b	44.92	135c	1.58	4573a	72.38	9	309	15	508
**5**	Pentanal	MS,RI,Std	715	935	732	935	19.25a	2.79	16.38a	1.74	9.81b	2.073	22	<1	<1	<1
**6**	Isopropyl alcohol	MS,RI,Std	510	884	510	884	7.8	1.63	— ^E^	—	—	—	6400	<1	—	—
**7**	1-Propanol	MS,RI,Std	536	1037	536	1037	137ab	11.42	116b	13.26	266a	23.48	200	<1	<1	<1
**8**	1-Butanol	MS,RI,Std	654	1142	654	1150	4.38a	1.56	2.54a	2.19	9.47a	2.73	130	<1	<1	<1
**9**	Hexanal	MS,RI,Std	802	1084	802	1084	47.28a	9.72	53.63a	8.89	33.56b	7.23	9	5	6	4
**10**	Dimethyl disulfide	FPD,RI,Std	785	1086	785	1086	1139a	48.9	56.32b	4.83	23.94b	2.04	7	163	8	3
**11**	2-Methyl-1-propanol	MS,RI,Std	628	1098	628	1094	489	49.13	—	—	1.59	3.048	640	<1	—	<1
**12**	(E)-2-Methyl-2-butenal	MS,RI,Std	743	1088	745	1088	19.32b	1.98	21.21b	1.89	231a	29.9	380	<1	<1	<1
**13**	Limonene	MS,RI,Std	1031	1192	1031	1198	45.79a	5.34	3.61b	1.34	1.98b	2.16	5.9	8	1	<1
**14**	(*E*)-2-Heptenal	MS,RI,Std	960	1334	961	1336	271a	25.27	2.84c	1.19	108b	9.94	550	<1	<1	<1
**15**	2-Butenal	MS,RI,Std	646	1050	644	1047	—	—	—	—	118	10.03	420	—	—	<1
**16**	2-Methylbutanol	MS,RI,Std	742	1206	742	1208	734c	49.8	1123b	32.3	3879a	54.73	140	5	8	28
**17**	3-Methylbutanol	MS,RI,Std	736	1209	732	1209	15.12b	1.23	26.54ab	2.89	38.95a	3.44	250	<1	<1	<1
**18**	2-Pentylfuran	MS,RI,Std	994	1239	994	1235	68.41a	7.99	10.34b	2.88	7.96b	2.66	270	<1	<1	<1
**19**	3-Octanone	MS,RI,Std	984	1243	984	1240	863b	21.36	6300a	78.29	950b	33.73	1.3	672	4846	731
**20**	1-Pentanol	MS,RI,Std	763	1262	763	1256	7.02a	1.82	6.84a	2.47	5.41a	2.37	4000	<1	<1	<1
**21**	Bis(methylthio) methane	FPD,RI,Std	898	1280	896	1282	5.76ab	1.63	7.89a	2.51	3.28b	3.29	0.012	480	658	273
**22**	2-Octanone	MS,RI,Std	965	1244	965	1244	6.73a	1.49	11.92a	1.38	9.64ab	3.79	50	<1	<1	<1
**23**	p-cymene	MS,RI,Std	1027	1295	1027	1295	2.73a	9.85	4.81a	2.39	0.18b	2.62	4	<1	1	<1
**24**	Octanal	MS,RI,Std	1006	1282	1006	1280	873a	19.1	42.9c	4.52	436b	33.29	0.7	1247	61	623
**25**	1-Octen-3-one	MS,RI,Std	975	1305	975	1305	719b	31.48	1034a	85.5	81.73c	7.48	0.8	899	1293	102
**26**	Isobutyl hexanoate	MS,RI,Std	1143	1356	1145	1356	2.14	1.19	—	—	—	—	3	<1	—	—
**27**	Nonanal	MS,RI,Std	1104	1358	1104	1358	65.38	7.36	—	—	38.75	3.16	40	2	—	<1
**28**	Heptanoic acid ethyl ester	MS,RI,Std	1089	1329	1093	1329	11.59ab	1.2	13.92a	1.22	7.83b	3.62	39	<1	<1	<1
**29**	1-Hexanol	MS,RI,Std	854	1364	851	1360	3.89ab	1.41	1.29b	1.23	4.38a	2.39	100	<1	<1	<1
**30**	3-Octanol	MS,RI,Std	991	1386	995	1386	322.76b	20.19	2157a	49.8	248.19b	21.29	22	15	98	11
**31**	Octanoic acid ethyl ester	MS,RI,Std	1196	1442	1196	1446	—	—	17.53	1.44	—	—	22	—	<1	—
**32**	(E)-2-Octenal	MS,RI,Std	1057	1432	1057	1434	—	—	7.68	1.63	2.19	2.19	3	—	3	<1
**33**	(E)- 2-Nonenal	MS,RI,Std	1161	1536	1161	1537	14.72ab	1.76	23.59a	2.48	8.76b	3.83	0.4	37	59	22
**34**	P-cresyl methyl ether	MS,RI,Std	1018	1445	1018	1445	870.84	20.73	—	—	—	—	560	2	—	—
**35**	1-Heptanol	MS,RI,Std	972	1457	972	1457	3.54a	1.66	1.47a	1.25	3.25a	2.26	200	<1	<1	<1
**36**	1-Octen-3-ol	MS,RI,Std	982	1426	982	1426	437b	42.8	5849a	57.69	566b	44.32	1	437	5849	566
**37**	3-(Methylthio)propanal	FPD,RI,Std	907	1456	907	1456	2.89ab	1.098	1.28a	1.42	4.93a	1.097	0.1	29	13	49
**38**	1-Octanol	MS,RI,Std	1068	1564	1068	1564	5.73a	1.48	3.66ab	1.38	2.14b	1.19	37	<1	<1	<1
**39**	Dimethyl sulfoxide	FPD,RI,Std	825	1560	820	1560	—	—	—	—	5.93	2.42	—	—	—	—
**40**	Benzaldehyde	MS,RI,Std	963	1528	963	1528	6.82a	3.53	0.97b	1.069	8.95a	2.74	320	<1	<1	<1
**41**	2-Methylbutanoic acid	MS,RI,Std	858	1650	858	1652	15.13ab	2.39	16.85a	1.77	11.72b	2.98	20	<1	<1	<1
**42**	3-Methylbutanoic acid	MS,RI,Std	846	1684	875	1686	8.87ab	2.65	9.45a	2.82	6.32b	3.51	1	9	9	6
**43**	2-Acetylthiazole	FPD,RI,Std	1015	1652	1015	1652	5.84a	2.47	4.71a	3.58	3.43a	1.27	4	1	1	<1
**44**	Benzeneacetaldehyde	MS,RI,Std	1051	1646	1051	1646	26.77b	2.74	23.94b	3.3	403a	45.39	0.7	38	34	576
**45**	Dipropyl trisulfide	FPD,RI,Std	1326	1659	1326	1659	7.52a	1.88	6.18a	2.67	5.57a	2.45	4.3	2	1	1
**46**	3-(Methylthio)propanol	FPD,RI,Std	998	1706	998	1706	18.19ab	2.56	11.84b	1.25	23.19a	1.79	500	<1	<1	<1
**47**	Benzyl alcohol	MS,RI,Std	1038	1890	1035	1886	—	—	0.97	0.088	—	—	100	—	<1	—
**48**	Dimethyl sulfone	FPD,RI,Std	923	1912	925	1912	—	—	5.88	1.43	—	—	—	—	—	—
**49**	Phenylethyl alcohol	MS,RI,Std	1110	1923	1113	1923	2.25c	1.29	21.67b	2.35	211a	37.89	80	<1	<1	3
**50**	gamma-nonalactone	MS,RI,Std	1370	2012	1370	2012	11.32a	1.43	9.54ab	2.93	8.73b	2.63	25	<1	<1	<1
**51**	[Bis(2-methyl-3-furyl)disulfide]	FPD,RI,Std	1425	2156	1425	2156	—	—	—	—	1.81	2.15	0.014	—	—	129

^A^ The volatile compounds detected in truffle samples. ^B^ The retention time of volatile compounds on DB-5 and HP-Innowax columns [20]. ^C^ MS: mass spectrum comparison using Wiley library; RI: retention index in agreement with literature value; Std: confirmed by authentic standards. ^D^ Values with different roman letters (a–c) in the same row are significantly different according to the Duncan test (*p* < 0.05). ^E^ not detected. ^F^ The threshold of volatile compounds referred to in the literature. ^G^ Detection odor threshold in air according to ref [29,30]. ^H^ The OAV of the compounds.

**Table 3:** Standard curves, validation range and correlation coefficients of standards (r^2^) for the volatile compounds in truffle samples.

No	Compound	Standard Curve	r^2^	Validation Range (μg kg^−1^)
**1**	Methanethiol	y = 0.065x − 0.0037	0.986	1–10
**2**	Dimethyl trisulfide	y = 1.7x + 0.0373	0.973	500–5000
**3**	2-Methylbutanal	y = 4.53x − 0.00591	0.981	50–500
**4**	3-Methylbutanal	y = 0.84x + 0.109	0.971	10–5000
**5**	Pentanal	y = 1.27x + 0.054	0.982	1–20
**6**	Isopropyl alcohol	y = 2.13x + 0.0016	0.993	1–10
**7**	1-Propanol	y = 3.39x − 0.0303	0.942	50–500
**8**	1-Butanol	y = 1.16x − 0.0239	0.996	1–10
**9**	Hexanal	y = 0.70x − 0.0531	0.971	10–100
**10**	Dimethyl disulfide	y = 4.53x − 0.591	0.968	10–2000
**11**	2-Methylpropanol	y = 0.99x − 0.0477	0.987	1–500
**12**	(*E*)-2-Methyl-2-butenal	y = 1.51x + 0.019	0.979	10–500
**13**	Limonene	y = 3.40x − 0.041	0.988	1–50
**14**	Heptenal	y = 1.56x − 0.0193	0.984	1–500
**15**	2-Butenal	y = 4.73x − 0.025	0.983	5–200
**16**	2-Methylbutanol	y = 0.91x + 0.031	0.986	500–5000
**17**	3-Methylbutanol	y = 1.05x + 0.076	0.985	5–50
**18**	2-Pentylfuran	y = 0.80x + 0.0492	0.971	5–100
**19**	3-Octanone	y = 0.38x + 0.0852	0.997	500–10000
**20**	1-Pentanol	y = 0.88x − 0.017	0.992	1–10
**21**	Bis(methylthio) mathane	y = 0.9x + 0.145	0.977	2–20
**22**	2-Octanone	y = 0.26 − 0.0138	0.993	2–20
**23**	4-Isopropyltoluene	y = 0.1277x + 0.00985	0.987	0.1–5
**24**	Octanal	y = 1.654x − 0.0235	0.973	20–1000
**25**	1-Octen-3-one	y = 1.977x + 0.0713	0.986	50–2000
**26**	Isobutyl hexanoate	y = 1.488x − 0.0790	0.982	0.1–5
**27**	Nonanal	y = 1.61x − 0.0233	0.985	10–100
**28**	Heptanoic acid ethyl ester	y = 2.87x − 0.0188	0.976	1–20
**29**	1-Hexanol	y = 1.70 − 0.0918	0.992	1–10
**30**	3-Octanol	y = 0.93x + 0.029	0.969	200–5000
**31**	Octanoic acid ethyl ester	y = 1.07 + 0.068	0.983	5–50
**32**	(E)-2-Octenal	y = 1.50x − 0.0218	0.979	1–10
**33**	(E)-2-Nonenal	y = 2.59x − 0.0376	0.986	2–50
**34**	P-cresyl methyl ether	y = 0.79 − 0.0034	0.967	20–200
**35**	1-Heptanol	y = 1.49x − 0.00208	0.983	1–10
**36**	1-Octen-3-ol	y = 2.13x − 0.0289	0.995	200–10000
**37**	3-(Methylthio)propanal	y = 2.48x + 0.0102	0.992	0.5–5
**38**	1-Octanol	y = 3.51x − 0.0472	0.992	1–10
**39**	Dimethyl sulfoxide	y = 0.30x − 0.0446	0.989	1–10
**40**	Benzaldehyde	y = 0.40x − 0.0595	0.976	0.5–10
**41**	2-Methylbutanoic acid	y = 0.78x − 0.0421	0.971	2–20
**42**	3-Methylbutanoic acid	y = 0.89x − 0.0086	0.975	1–10
**43**	2-Acetylthiazole	y = 1.25x + 0.41	0.965	1–10
**44**	Benzeneacetaldehyde	y = 1.77x − 0.0142	0.982	20–500
**45**	Dipropyl trisulfide	y = 2.40x − 0.0320	0.982	2–10
**46**	3-(Methylthio)propanol	y = 2.072x − 0.0282	0.979	2–20
**47**	Benzyl alcohol	y = 2.47x + 0.0079	0.987	0.05–1
**48**	Dimethyl sulfone	y = 1.97x + 0.031	0.981	1–10
**49**	Phenylethyl alcohol	y = 4.95x − 0.0356	0.973	2–500
**50**	Γ-Nonalactone	y = 0.78x + 0.0512	0.988	2–20
**51**	[Bis(2-methyl-3-furyl) disulfide]	y = 2.97x + 0.0053	0.976	0.2–2

**Table 4:** The mean intensity values of the seven attributes for the three truffles in descriptive sensory evaluation.

Sensory Attributes	Mean Score		
	T1	T2	T3
mushroom	6.27b ^A^	9.39a	6.03b
nutty, malty	3.14b	2.37c	4.37a
fatty, green	5.89a	4.19b	4.98ab
floral, sweet	0.98b	0.76b	3.39a
sulfury, musty	4.12a	4.23a	4.18a
roasted potato	2.07ab	1.34b	2.89a
rotten cabbage, corn	8.13a	7.96a	7.89a

^A^ Values with different roman letters (a–c) in the same row are significantly different according to the Duncan test (*p* < 0.05).

## Supplementary Materials

The following are available online, Figure S1: GC-MS chromatograms (T1 sample); Figure S2: GC-MS chromatograms (T2 sample); Figure S3: GC-MS chromatograms (T3 sample).
