# Supplementary material for: Characterization of the Key Aroma Compounds in Three Truffle Varieties from China by Flavoromics Approach
Source: Molecules. 2019 Sep 11;24(18):3305. doi: 10.3390/molecules24183305 (PMC6767217; doi:10.3390/molecules24183305)
Supplement: Supplementary file 1 [file molecules-24-03305-s001.pdf]

Supplementary material

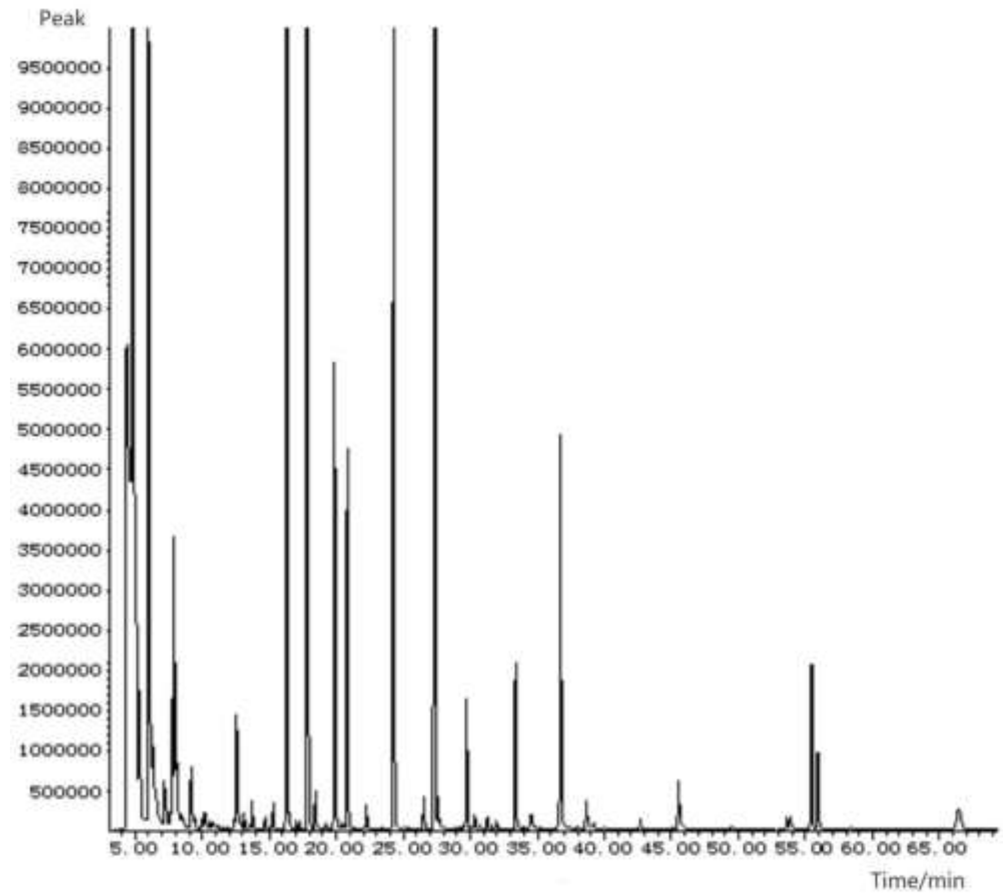

Fig 1. GC-MS chromatograms (T1 sample)

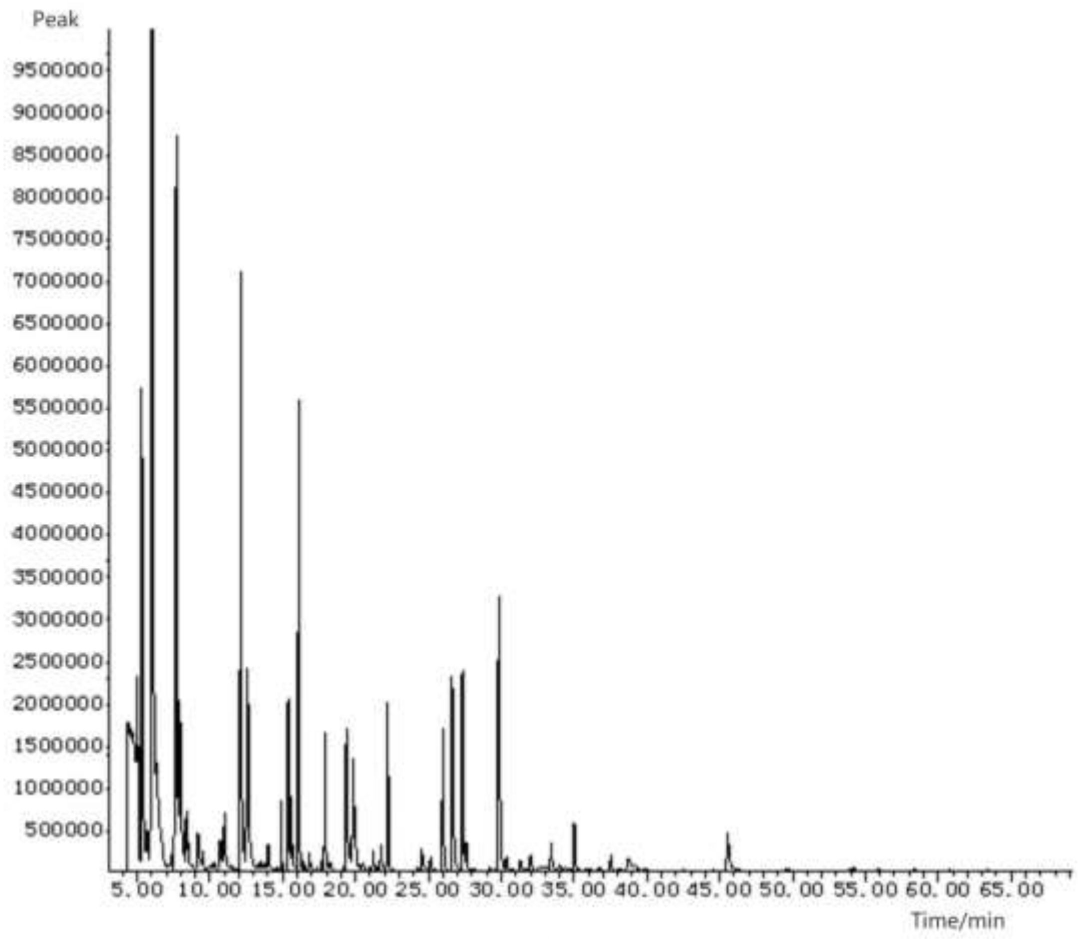

Fig 2. GC-MS chromatograms (T2 sample)

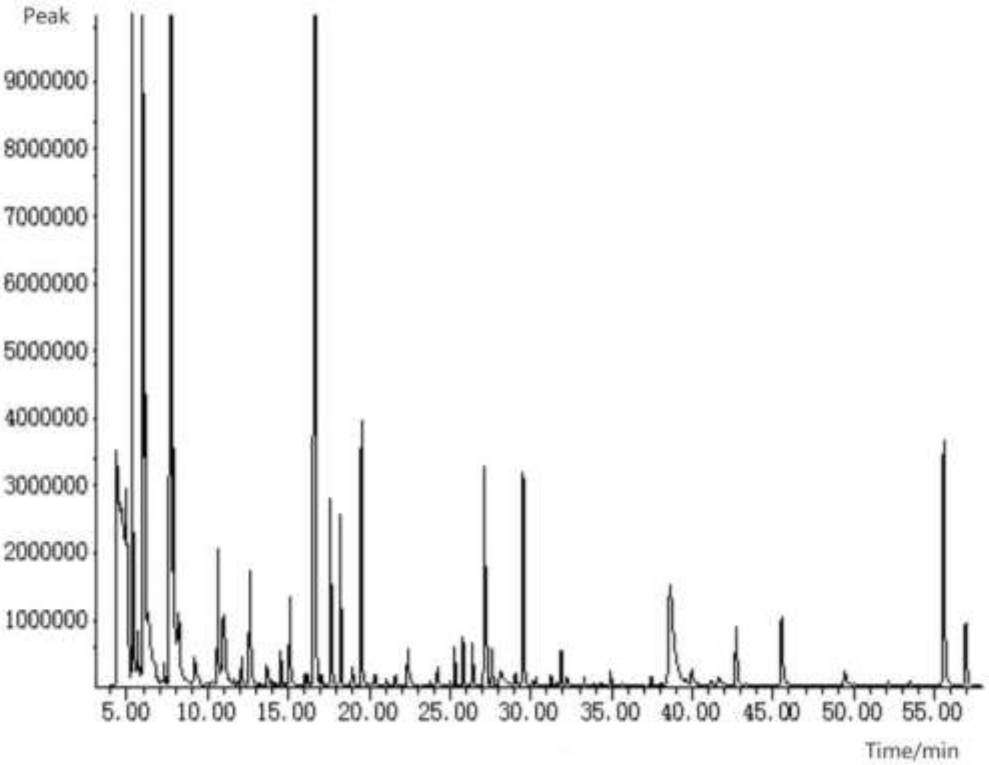

Fig 3. GC-MS chromatograms (T3 sample)
